# Predicting grey-sided vole occurrence in northern Sweden at multiple spatial scales

**DOI:** 10.1002/ece3.827

**Published:** 2013-10-06

**Authors:** Magnus Magnusson, Arvid Bergsten, Frauke Ecke, Örjan Bodin, Lennart Bodin, Birger Hörnfeldt

**Affiliations:** 1 Department of Wildlife, Fish and Environmental Studies, Swedish University of Agricultural SciencesUmeå, Sweden; 2 Stockholm Resilience Center, Stockholm UniversityStockholm, Sweden; 3 Department of Aquatic Sciences and Assessment, Swedish University of Agricultural SciencesUppsala, Sweden; 4 Institute of Environmental Medicine, Karolinska InstitutetStockholm, Sweden

**Keywords:** Boreal forest, connectivity, conservation, forest patch size, grey-sided vole, *Myodes*, population ecology, small mammals, stone fields

## Abstract

Forestry is continually changing the habitats for many forest-dwelling species around the world. The grey-sided vole (*Myodes rufocanus*) has declined since the 1970s in forests of northern Sweden. Previous studies suggested that this might partly be caused by reduced focal forest patch size due to clear-cutting. Proximity and access to old pine forest and that microhabitats often contains stones have also been suggested previously but never been evaluated at multiple spatial scales. In a field study in 2010–2011 in northern Sweden, we investigated whether occurrence of grey-sided voles would be higher in (1) large focal patches of >60 years old forest, (2) in patches with high connectivity to surrounding patches, and (3) in patches in proximity to stone fields. We trapped animals in forest patches in two study areas (Västerbotten and Norrbotten). At each trap station, we surveyed structural microhabitat characteristics. Landscape-scale features were investigated using satellite-based forest data combined with geological maps. Unexpectedly, the vole was almost completely absent in Norrbotten. The trap sites in Norrbotten had a considerably lower amount of stone holes compared with sites with voles in Västerbotten. We suggest this might help to explain the absence in Norrbotten. In Västerbotten, the distance from forest patches with voles to stone fields was significantly shorter than from patches without voles. In addition, connectivity to surrounding patches and size of the focal forest patches was indeed related to the occurrence of grey-sided voles, with connectivity being the overall best predictor. Our results support previous findings on the importance of large forest patches, but also highlight the importance of connectivity for occurrence of grey-sided voles. The results further suggest that proximity to stone fields increase habitat quality of the forests for the vole and that the presence of stone fields enhances the voles' ability to move between nearby forest patches through the matrix.

## Introduction

Globally, most forest landscapes have been actively used and managed resulting in fragmentation of natural habitats for many forest living species (Lindenmayer and Franklin 2002). The forest landscape in northern Sweden is heavily fragmented with a steady decline in amount of old forests due to selective cutting before the 1950s (Axelsson and Östlund 2001) and large-scale clear-cutting after the 1950s (Esseen et al. 1997; Ecke et al. 2013). Many threatened species are habitat specialists and should be negatively affected by habitat loss, fragmentation, and degradation as they benefit from environments that are relatively homogenous as predicted by the niche evolution theory (see e.g., Kerbiriou et al. 2009). It has been suggested that habitat specialists are declining throughout the world (Devictor et al. 2008). A possible explanation is that specialist species prefer the most stable sites and generalist species the more unstable ones subjected to environmental change (Julliard et al. 2006; see also Bergsten et al. 2013). In such situation, generalist species adapt to environmental changes, such as forest management, faster and can often be more successful competitors than habitat specialists (Bengtsson et al. 2000), and it has been shown that decreasing human disturbance is positively related to the number of specialist species (Kitahara and Fuji 1994).

**Figure 1:**
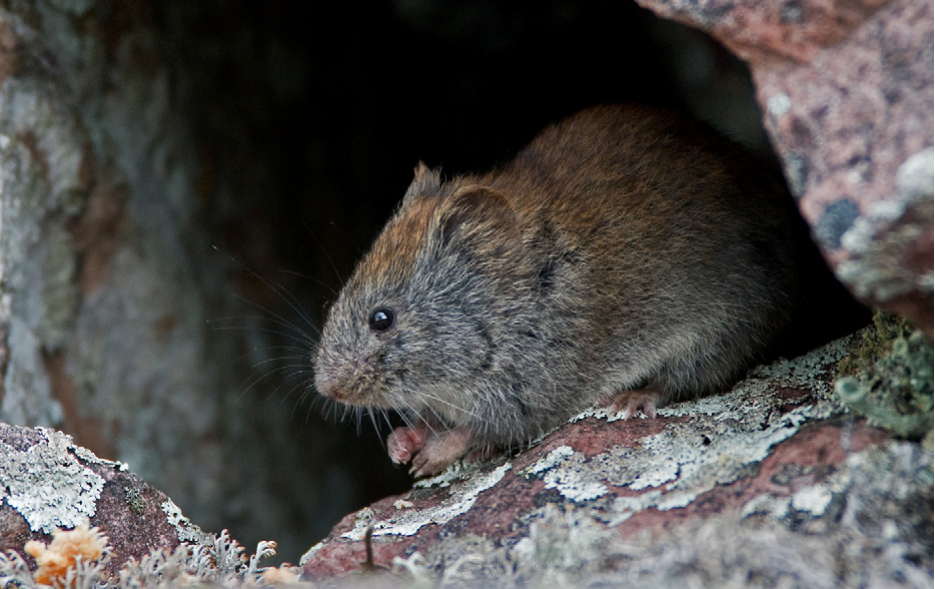
Grey-sided vole individual in a stone hole habitat (Photo credit: Rolf Segerstedt).

In lowland forests of Sweden, the grey-sided vole (*Myodes rufocanus*; Fig. 1), which has been described as primarily a forest living species in Scandinavia (Henttonen et al. 1992), can be treated as a specialist species as it seems to avoid clear-cuts (Christensen and Hörnfeldt 2006) and prefer larger, continuous forests (Ecke et al. 2006; Christensen et al. 2008). The species currently has much higher population numbers (> threefold in spring) in less fragmented near-mountainous forests compared with lowland forests (Ecke et al. 2010; Hörnfeldt 2012). A long-term decline of the grey-sided vole in lowlands forests of northern Sweden has been detected by the Swedish environmental monitoring program (see Hörnfeldt 1994, 2004, 2012). The decline was thought to be a combination of mainly (1) fragmentation and habitat loss of forest habitats in the forest landscape (Hörnfeldt 2004; Ecke et al. 2006, 2010; Hörnfeldt et al. 2006; Christensen et al. 2008) and (2) a changing climate with warmer winters leading to decreased snow cover quality with increased ice-formation on the ground, which affects winter survival of small mammals negatively (see e.g., Lindström and Hörnfeldt 1994; Ims et al. 2008; Kausrud et al. 2008).

Age of forest, focal forest patch size, proximity, and access to old pine forest are variables associated with the occurrence of grey-sided voles in lowland forests (Christensen and Hörnfeldt 2006; Ecke et al. 2006, 2010; Hörnfeldt et al. 2006). Previous studies have suggested that occurrences are strongly associated with patches of old forest >80 ha (Ecke et al. 2006, 2010), but connectivity of forest patches has not previously been addressed using recent connectivity measures such as the integral index of connectivity (IIC; Pascual-Hortal and Saura 2006). A study in subalpine southern Norway showed that preferred microhabitats for grey-sided voles often contain stones (boulders; Johannesen and Mauritzen 1999) as they may provide shelter from predation. However, the proximity of stone fields for the occurrence of grey-sided voles has not been investigated in detail at the landscape scale. Also, as we have information on habitat requirements (Christensen and Hörnfeldt 2006; Ecke et al. 2006, 2010; Hörnfeldt et al. 2006), it is also possible to use least-cost path analysis (see e.g., Verbeylen et al. 2003) to simulate the least-cost distance the vole moves between stone fields and forest patches.

The aim of this study is to explore the dependence of occurrence and density of *M. rufocanus* on habitat properties at different spatial scales: Firstly, we investigate whether habitat properties at the microhabitat scale can help explain occurrence. Secondly, we explore the effect of habitat properties at the local scale on occurrence and density. Thirdly, on the landscape scale, we analyzed the dependence of occurrence of *M. rufocanus* related to focal forest patch size, connectivity of forest patches, and proximity to stone field components using Euclidean and least-cost path distance to stone field components.

## Methods

### Mapping of habitat patches

**Figure 2:**
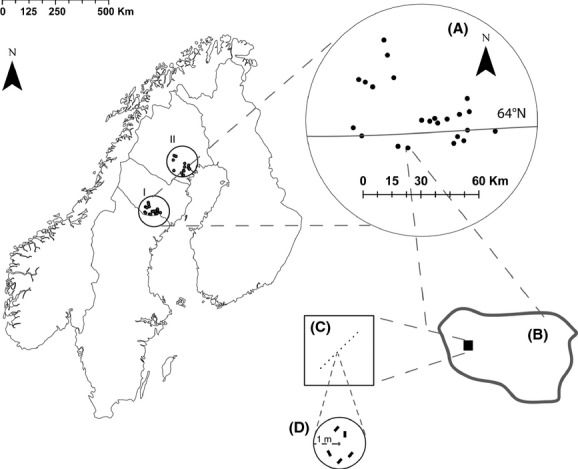
Study areas (circles) located in Västerbotten (I) and Norrbotten (II) county, northern Sweden. In Västerbotten, 23 study sites were surveyed (A) and in Norrbotten 16 sites. Each site representing a 1-ha square was randomly placed within a > 60 years old forest patch (B). The surveyed line transect (representing the sampling plot) consisted of 10 trap stations centered along a diagonal (C) with five snap traps placed within a circle with a 1 m radius at each trap station (D).

The selection of forest patches was carried out in two steps: Firstly, two old pine dominated landscapes in northern Sweden were selected (see Fig. 2). Old pine forest has previously been identified as important for grey-sided vole occurrence (see Ecke et al. 2006). Secondly, mapping of patches of forest >60 years old was carried out using the spatial analyst tools in ArcGIS 9.3 (ESRI 2012) and *k*NN-Sweden 2005 (Anonymous 2010). Later, the forest landscape was remapped using a segmented raster of *k*NN-Sweden 2000 (Anonymous 2008; for segmentation procedure see Hagner 1990). Forest patches clear-cut after 2000 until 2010 were reclassified as clear-cuts in the study areas using spatial data from the Swedish Forest Agency as our survey was carried out in 2010 and 2011. The *k*NN-Sweden map project combines Swedish National Forest inventory data with satellite images (see Reese et al. 2003). There is a large uncertainty and systematic underestimation of old forest in the *k*NN-Sweden data (Reese et al. 2003). 100-year-old trees often have an age of 80–90 year in the *k*NN data, and areas should preferably be large to give a reasonably good accuracy (Reese et al. 2003). We choose to focus on patches of >60-year-old forest as these forests have been spared from large-scale clear-cutting practices that started in the 1950s in northern Sweden and Ecke et al. (2010) also used forest patches >60 years when studying grey-sided vole dependence on forest patch size at a landscape scale. Further, the accuracy on a stand level for age 50–69 years is good with a mean error of ∼10 years (Reese et al. 2003).

### Mapping of connectivity of focal forest patches

The integral index of connectivity (IIC; Pascual-Hortal and Saura 2006) estimates the *habitat reachability* in a landscape based on the maximum Euclidean dispersal distance of a focal species. By calculating how much IIC decreases following a hypothetical loss of any habitat patch, the relative importance of a focal patch (dIIC) in a habitat network is estimated from the size of the focal patch and the potential for species movement to and from habitat areas elsewhere in the landscape. dIIC consists of three fractions that correspond to different ways in which a patch can contribute to the total habitat availability in the habitat network; namely, the intra, flux, and connector fractions (Saura and Rubio 2010). In our analysis, we omit the intrafraction as it is fully independent on how the focal patch is connected to other patches and because it has a perfect monotonic relationship with the focal patch area, which our analysis already accounts for (Saura and Rubio 2010). Also the connector fraction is excluded as it does not predict colonizations in the focal patch, but only measures the contribution of the focal patch to connectivity between other patches (Bodin and Zetterberg 2010; Saura and Rubio 2010; Gil-Tena et al. 2013). Finally, we divide the remaining flux fraction with the area of the focal patch (cf. Laita et al. 2010). Thus, we avoid the strong collinearity between the flux fraction and the focal patch area, enabling a better evaluation of the predictors and their possible redundancies in the analysis (where the effect of focal patch area on vole occurrence is controlled for separately). As a result, we do not estimate the contribution of focal patches to the total habitat availability in the landscape as in the original formulation of IIC (Pascual-Hortal and Saura 2006). Rather, we estimate the effect of potential flux given the topological proximity and habitat area of other forest patches in the study area.

For a measure of dispersal ability of the grey-sided vole, it could be plausible to use the distance of around 0.2 km reported by Saitoh (1995) for natal dispersal as well as the long-range dispersal of about 3 km for adults in the increase phase of the vole cycle reported by Oksanen et al. (1999). Furthermore, a calculation based on relationships between home range size and dispersal distance as found by Bowman et al. (2002), assuming a home range size of 0.135 ha for reproducing grey-sided vole females (Löfgren 1995), gives a median dispersal distance of 0.26 km and a maximum dispersal distance of 1.47 km. Thus, there is a range of plausible dispersal distance estimates of relevance to choose from. To test the effect of different dispersal assumptions, the maximal dispersal distance for calculation of connectivity (see above) was, in this study, accordingly set to 250, 500, 1000, 1500, 2000, and 3000 m, which are all within the range of cited dispersal distances.

### Selection of focal forest patches in two study areas

Christensen et al. (2008) proposed that future studies of occurrence and density of *M. rufocanus* would be suitable to do in pristine areas (that is found in some national parks and nature reserves) of various size and forest composition. Thus, a range of small to large patches (range: 2.6 ha–2370 ha) were selected, preferably within protected areas. Firstly, all patches were roughly categorized based on their area (small, large) and isolation (low, high connectivity) using 2005 *k*NN data (Anonymous 2010) and whether they belonged to a protected area. Secondly, a number of patches for each size and isolation category were selected from the resulting list aiming to represent the most extreme combinations of large and small patches with high or low isolation. The selection resulted in 16 forest patches (all in protected areas) in Norrbotten county (II in Fig. 2) and 24 patches (16 in protected areas and eight patches outside protected areas) in Västerbotten county (I in Fig. 2) that were trapped in autumn 2010 and 2011. Both these years had high population densities of grey-sided vole and represented a double peak (two consecutive years with high population numbers) in the vole cycle according to the National environmental monitoring program near Umeå in northern Sweden (cf. Hörnfeldt 2012). We judged that fall trapping from these 2 years would be comparable as the cyclic decline in density was only pronounced during the following winter 2011/2012, resulting in very low numbers in spring 2012 (cf. Hörnfeldt 2012).

All trappings were made with snap traps following the method used in the National environmental monitoring program of Sweden (see Hörnfeldt 1978, 1994, 2004, 2012), that is, for three consecutive nights using a 90-m-long transect with 10 trap stations along one of the diagonals of a 1-ha sampling plot (see IV, V and VI in Fig. 2). In each patch of >60-year-old forest, a randomly selected 1-ha sampling plot was chosen, with the criteria that the whole transect inside the plot should run through the forest. Note, however, that we were unable to account for potentially important local ground characteristics (see introduction) in the selection of sampling plots, as we were not aware of maps with that type of information.

### Microhabitat inventories

After we finalized the trappings in the selected sites, a microhabitat inventory was undertaken in both study areas (Västerbotten, I and Norrbotten, II) to investigate forest and ground characteristics of potential importance to the grey-sided vole. The method is similar to the one applied by Ecke et al. (2001, 2002). In ten quadratic plots, with 2.5 m sides, 10 m apart and centered on the 10 trap stations (see above) in each transect, vegetation and structural variables were estimated. The following variables were measured according to a 5-graded scale ((1) 0% (2) >0–12% (3) >12–25% (4) >25–50% (5) >50%; for definition of variables see Table 1): Tree layer 1 and 2, bushes, tree lichens, fine woody debris (FWD), stones, large stones, umbrella vegetation, field layer vegetation, mosses, lichens, bilberry, cowberry, and grasses. Large stones and snags in each plot were counted. Coarse woody debris (CWD) was measured as the total length of logs in each plot. Number of large (ø > 5 cm) and small (ø < 5 cm) holes were sorted into the following classes: (0) = 0–4 holes, (5) = 5–9 holes, (10) = 10–19 holes, (20) = 20–39 holes, (40) = ≥40 holes. Large stone holes (i.e., holes beneath stones) were sorted into the same classes with a slight modification of the first class into two classes: (0) = 0 holes, (1) = 1–4 holes, as a trapping site with merely one large hole beneath a large stone could in theory provides a positive contribution for grey-sided voles. Tree species composition was measured by ocular estimation of the proportion of each tree species within ∼ 20 m distance from the middle of the trap station.

**Table 1:** Description and abbreviation of the structural habitat variables used for characterization of trap stations in the study

Variable	Abbreviation	Description
Bilberry	Bilb	Cover of bilberry dwarf shrubs^1^
Cowberry	Cowb	Cover of cowberry dwarf shrubs^1^
Tree lichens	T-lichens	Cover of lichens that grows on trees^1^
Coarse woody debris	CWD	Length (m) of coarse woody debris (ø ≥ 10 cm)
Fine woody debris	FWD	Cover of fine woody debris (ø < 10 cm) 1
Snags	Snags	Number of snags in the inventory plot (no scale)
Lichens	Lichens	Cover of ground lichens^1^
Mosses	Mosses	Cover of mosses^1^
Grasses	Grasses	Cover of grasses^1^
Umbrella vegetation	U-veg	Cover of umbrella vegetation (height >50 cm) 1
Field layer vegetation	Fl-veg	Cover of field layer vegetation (height <50 cm) 1
Large stones	Lstones	Cover of large stones (d > 50 cm)^1^
Number of large stones	Lstones (no)	Number of large stones in the inventory plot (d > 50 cm; no scale)
Stones	Stones	Cover of visible stones (d > 10 cm) 1
Small holes	S-holes	Small holes (ø < 5 cm) 2
Large holes	L-holes	Large holes (ø > 5 cm) 2
Large stone holes	LS-holes	Large holes beneath stones (ø > 5 cm) 3
Prop. of pine	Pine	Prop. of pine trees^4^
Prop. of spruce	Spruce	Prop. of spruce trees^4^
Prop. of birch	Birch	Prop. of birch trees^4^
Prop. of aspen	Aspen	Prop. of aspen trees^4^
Prop. of goat willow	G-willow	Prop. of goat willow trees^4^
Prop. of other deciduous trees	Other trees	Prop. of other deciduous trees^4^
Bushes	Bushes	Cover of the bush layer (height = 0.5 m–5 m) 1
Tree layer 1	TL1	Cover of upper tree layer (height ≥5 m)^1^
Tree layer 2	TL2	Cover of the lower tree layer (height = at least 5 m lower than the upper tree layer)^1^

1 Cover (%); 1 = 0%, 2 ≥ 0–12%, 3 ≥ 12–25%, 4 ≥ 25–50%, 5 ≥ 50%.

2 Number of small and large holes; 0 = 0–4 holes, 5 = 5–9 holes, 10 = 10–19 holes, 20 = 20–39 holes, 40 ≥ 40 holes.

3 Number of large stone holes; 0 = 0 holes, 1 = 1–4 holes, then the same categories as for S- and L-holes.

4 Proportion (%) of trees in >60-year-old forest within 20 m from each trap station.

In Västerbotten (study area I), we also obtained quaternary deposits geology data on the landscape scale from the Swedish Geological Survey (Stendahl et al. 2009). For our purpose, we defined stone fields as both open and forested areas with pronounced amount of large stones in the ground layer. Using the stone field data, we analyzed least-cost path distances for study plots with and without grey-sided voles to the nearest stone field within a stone component. A stone component we define as (similar to a network component) made up of nodes, in this case stone fields, connected with lines. Network components are described in detail in fig. 3 in Bodin et al.(2006) and we used the ArcGIS tool package Matrix Green (Bodin and Zetterberg 2010) for this analysis. The stone fields that make up a stone component are all located within a radius of, in this study, 1000 m from each other, and the requirement for including a stone field component in our analysis was that the component should be in total >100 ha large. In the least-cost path analyses, a cost surface was created from pixel data in *k*NN 2010 (Anonymous 2012) and the Swedish land cover map (Lantmäteriet 2005) using clear-cuts 0–20 years old and lakes as hindrance to movement and old pine forest >100 years as facilitating movement of the vole based on previous knowledge about landscape elements related to grey-sided vole occurrence (see Ecke et al. 2006). To model the most effective paths between study plots and stone field components, the cost distance tool in the Spatial Analyst extension tools in ArcGIS 9.3 was used (ESRI 2012). In the cost distance tool, the cost of moving through each cell in the cost surface is taken into account and calculates the minimal cost to reach a given spot in the landscape (i.e., a sampling plot) from a source patch (i.e., a stone field). For a more detailed description of a cost distance analysis see Verbeylen et al. (2003).

### Statistical analyses

#### Dependence of habitat properties on microhabitat scale

For prediction of grey-sided voles by microhabitat characteristics, a mixed effect hierarchical model was applied using the MultiModel Inference package (MuMIn) in R 2.15 (R Development Core Team 2008). Firstly, we used the dredge function in the MuMIn package for model selection with all possible combinations according to the AICc –information criterion (AICc is used for small samples) with binomial errors and a logit link function and secondly, the model averaging function (mod.avg). Burnham and Anderson (2002) suggested that models with delta AICc >4 had considerably less empirical support. Following this suggestion, we present a table including models with ΔAICc <4. The sampling plots in the forest patches were treated as random effects while the individual 10 trap stations inside every forest patch were nested (hierarchical). Before model selection, collinearity among 25 variables was evaluated by excluding all independent variables that had a correlation value >0.5 (Spearman's correlation test). Then, all variables with low variance in the data (e.g., <1 in variance on a 1–5 scale) were discarded as these variables will have low influence on the result in the prediction model.

#### Dependence of habitat properties on local and landscape scale

Nonparametric Fisher exact test and Mann–Whitney *U*-test in Statistica 10 (StatSoft, Inc. 2011) were used for testing differences of patch size, connectivity and distance to stone fields from sampling plots with and without grey-sided voles.

Analyses of grey-sided vole densities (log transformed) in focal forest patches against (1) mean number of stone holes (log transformed); (2) mean proportion of pine forest >60 years (arc sin transformed); (3) forest patch size >60 years (log transformed) and (4) connectivity of forest patches (log transformed; 250 m–3 km) were made with scatterplots and fitting of a linear function that was tested with a Pearson correlation test.

A supplementary analysis to examine the relative importance of these landscape variables (focal patch area, connectivity and least-cost distance to stone fields) for predicting the occurrence of grey-sided voles was performed by Quadratic Discriminant Analysis (QDA), which is a generalization of Linear Discriminant Analysis (LDA; Mc Lachlan 1992). As with LDA the quadratic form seeks to classify objects into the group where the object's posterior probability of group affiliation is maximized. The posterior probability is formed as a function of the known (or assumed) prior probability for group affiliation combined with a function of the observed structural habit characteristics. Both LDA and QDA assume that the observations come from a multivariate normal distribution and the difference between the two methods is that LDA assumes that the covariance matrices for the structural habit characteristics in the two groups (see below) are equal, whereas QDA does not require this.

To evaluate the results of QDA, three outcomes will be used here. The first is referred to as specificity which estimates the probability of correctly classifying an object as belonging to the group MR = 0 (absence of *M. rufocanus*) is estimated. The second is referred to as sensitivity which estimates the probability of correctly classifying an object to group MR = 1 (presence of *M. rufocanus*). Finally, the total classification error rate is estimated.

The computations were performed with prior probabilities equal to the relative group sizes. The structural habitat characteristics were examined, alone and in combination with each other. To perform some test of the external validity of the classification function a “leave-one-out” computation, also referred to as jack-knife estimation, was carried out. Here the computations were repeated but for each object the classification functions were calculated based on the structural habitat characteristics of all other objects but the examined object. A large deviation between the outcomes for the complete analysis and the “leave-one-out” analysis is an indication of low external validity. The computations were carried out with the software STATA, version 12 (StataCorp 2011). All GIS-analysis were made using ArcGIS 9.3 and 10.x (ESRI 2012) except for connectivity analyses where Conefor 2.2–2.6 (Saura and Torné 2009) was used.

## Results

**Figure 3:**
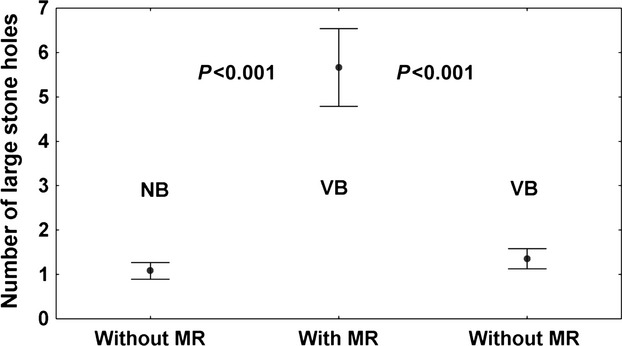
Number of large stone holes (mean ± SE, Ø > 50 cm) for trap stations without *M. rufocanus* in Norrbotten (*n* = 160) and with (*n* = 59) and without (*n* = 164) *M. rufocanus* in Västerbotten. The *P*-values denote significance levels for Mann–Whitney *U*-tests between trap stations with MR in Västerbotten and without MR in Västerbotten and Norrbotten, respectively.

In the Norrbotten study area (II in Fig. 2) no grey-sided voles were trapped in autumn 2010 in the 16 sampling plots. In autumn 2011, another site within study area II, where grey-sided voles occurred in the 1990s (cf. Ecke et al. 2002), was re-trapped during one night showing that the species was still present but suggesting that it was rare in that study area. In the Västerbotten study area (I in Fig. 2), the grey-sided vole was found in 16 of the 23 sampling plots in 2010 and 2011 (115 animals in total). Note, that after applying the segmented *k*NN2000-dataset prior to analysis (see material and methods), two sampling plots in Västerbotten in two separate forest patches were merged into one resulting in a total of 23 sampling plots. The local forest structure inventory revealed a potentially important difference in ground conditions between sampling plots in the two regions, as large stone holes were more common on sites with grey-sided vole in Västerbotten (I) than without voles in Norrbotten (II) (Fig. 3). In addition, according to data obtained from the Swedish Geological Survey, average distance to the nearest stone field component was significantly shorter (*P* < 0.01) for sampling plots with grey-sided vole in study area I (*n* = 16; 1122 ± 448; Mean ± SE) than without voles in II (*n* = 9; 3649 ± 846; Mean ± SE). In study area II, only nine of 16 sampling plots were covered by the geological data. Hereafter, we focus on study area I unless stated otherwise. Thirteen of sixteen (81%) study plots with voles were situated within 1500 m from stone field components. In contrast, none of seven plots without voles were within 1500 m from the stone field components (Table 2). Further, both the Euclidean and least-cost path distance to stone field components were significantly shorter for plots with than without voles (Table 2).

**Table 2:** Proportion of sampling plots within 1500 m, Euclidean distance and least-cost path distances from plots to stone field components for plots with (*n* = 16) and without (*n* = 7) *M. rufocanus* in Västerbotten. *P*-values denote significant differences between 1-ha sampling plots with and without *M. rufocanus* as tested with Fisher exact test (1) and Mann–Whitney *U*-test (2–3)

	Sampling plots		
		
Variable	With MR (*n* = 16)	Without MR (*n* = 7)	Significance
1. Proportion of sampling plots within 1500 m from stone field components (%)	81	0	*P* < 0.001
2. Euclidean distance (m) from sampling plots to nearest stone field component (mean ± SE)	1122 ± 448	3991 ± 537	*P* < 0.01
3. Least-cost path distance (m) from sampling plots to nearest stone field component (mean ± SE)	1299 ± 514	4479 ± 699	*P* < 0.01

### Microhabitat and local scale

*Occurrence* of grey-sided voles at the trap station level were best predicted in the current study area I by a combination of a high proportion of >60-year-old pine forest around the trap station and high number of large holes (>5 cm in diameter) beneath stones (i.e., “large stone holes”; Table 3b). These two variables had the highest possible importance values of 1.0 in the mixed effect logit model run for microhabitat characteristics using all trap stations in study area I (*n* = 223). Both variables had a positive direction and were included in all models with a ΔAICc<4, and both were significant in model-averaged directions (*P* < 0.01). We choose to present all models with ΔAICc<4 as Burnham and Anderson (2002) states that models with ΔAICc>4 have considerably less empirical support. The null model had a ΔAICc value of 12.47 (Table 3a). Small holes (<5 cm) had an importance value of 0.7, a positive direction (0.05) but was only marginally significant (*P* = 0.053). The rest of the variables had less predictive power, but it is interesting to note that the preferred food plant, bilberry had a negative direction (−0.16).

**Table 3:** General linear mixed effect regression models with logit link for occurrence of grey-sided voles at trap stations (*n* = 223) nested in patches of >60 years old forest (*n* = 23) in study area I in the inland of Västerbotten county, northern Sweden. (A) The null model followed by all models with Δ_*i*_ < 4 are included in the explanatory variables table. Explanatory variables included AIC_*c*_ (smaller values indicate a better fit to the data), Δ_*i*_ (difference in AIC_*c*_ between model *i* and the model with the smallest AIC_*c*_), *w*_*i*_, (Akaike weight). (B) Full model-averaged directions, sorted with decreasing relative importance values (ranging from 0–1, 1 being highest) and significance levels for (Pr ≥ │z│) each variable are given

Explanatory variables	AIC_*c*_	Δ_*i*_	*w*_*i*_
(A)			
(Intercept)	210.70	12.47	0.00
(Intercept) + Bilb + Pine + LS-holes + S-holes + TL1	198.26	0.00	0.14
(Intercept) + Pine + LS-holes + S-holes	198.50	0.24	0.12
(Intercept) + Bilb + Pine + LS-holes + S-holes	198.55	0.29	0.12
(Intercept) + Pine + LS-holes + S-holes + TL1	198.61	0.35	0.11
(Intercept) + Pine + LS-holes + TL1	199.62	1.36	0.07
(Intercept) + Pine + LS-holes	199.83	1.56	0.06
(Intercept) + Bilb + CWD + Pine + LS-holes + S-holes + TL1	199.92	1.66	0.06
(Intercept) + Bilb + Pine + LS-holes + TL1	199.95	1.69	0.06
(Intercept) + Bilb + CWD + Pine + LS-holes + S-holes	200.09	1.83	0.05
(Intercept) + CWD + Pine + LS-holes + S-holes	200.23	1.97	0.05
(Intercept) + CWD + Pine + LS-holes + S-holes + TL1	200.46	2.20	0.05
(Intercept) + Bilb + Pine + LS-holes	200.50	2.24	0.04
(Intercept) + CWD + Pine + LS-holes + TL1	201.72	3.46	0.02
(Intercept) + CWD + Pine + LS-holes	201.89	3.63	0.02
(Intercept) + Bilb + CWD + Pine + LS-holes + TL1	202.05	3.79	0.02

Variable	Full model-averaged directions	Relative variable importance	Significance of model-averaged directions
(B)			
Pine	2.610	1.00	0.001
LS-holes	0.143	1.00	0.005
S-holes	0.052	0.70	0.053
TL1	0.156	0.53	0.132
Bilb	−0.164	0.49	0.147
CWD	−0.016	0.28	0.608

**Figure 4:**
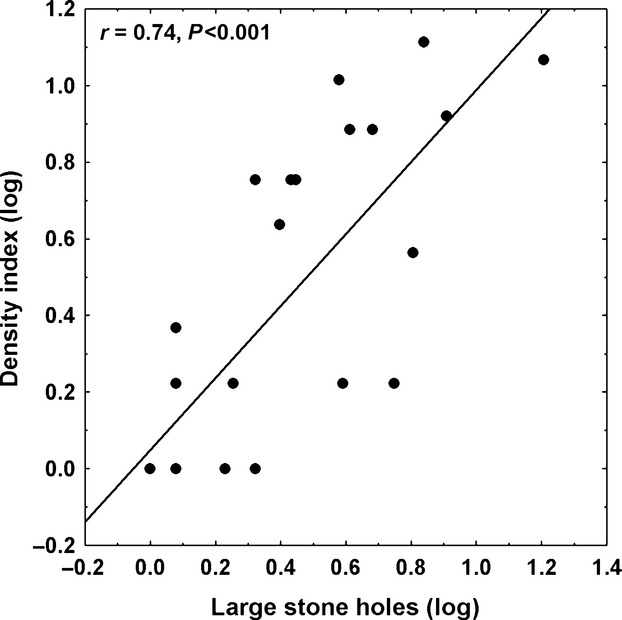
Density index for *M. rufocanus* (no. of voles/100 trap nights) against mean number of stone holes in each of the surveyed sampling plots (*n* = 23) in study area I. The scatter plot is fitted with a linear function and the *P*-value denote the significance of the correlation coefficient, *r*.

The sampling plots in Västerbotten showed a significant positive correlation between *density* (no. of individuals per 100 trap nights) of grey-sided voles and mean number of large stone holes (Fig. 4; *r* = 0.74; *P* < 0.001). Similarly, proportion of >60-year-old pine forest within sampling plots also seemed to predict grey-sided vole density but with a lower *r*-value (*r* = 0.61; *P* < 0.01).

### Landscape scale

**Figure 5:**
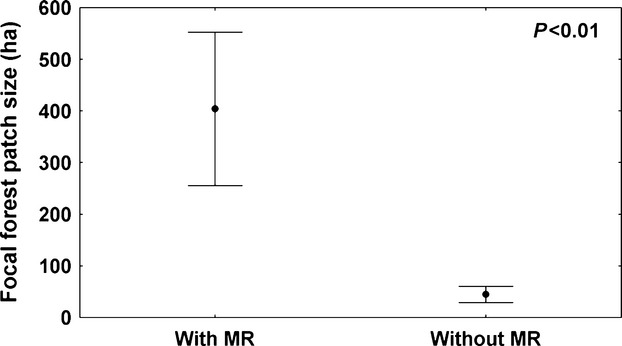
Focal forest patch size (mean ± SE) for patches with >60-year-old forest with (*n* = 16) and without (*n* = 7) *M. rufocanus* in study area I. The *P*-value denotes the significance level for a Mann–Whitney *U*-test of the difference between groups.

**Figure 6:**
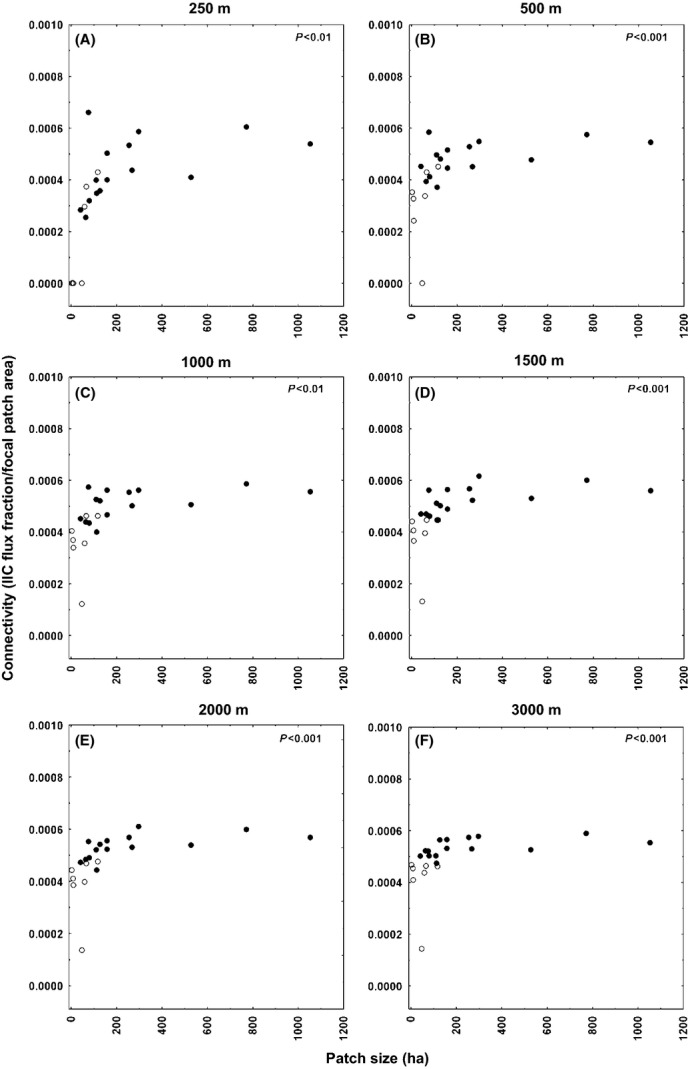
Scatter plots of connectivity for focal patches with >60-year-old forest (dIICflux/area) against focal patch size of >60-year-old forest (ha) based on assumed dispersal distance of 250 m (A), 500 m (B), 1000 m (C), 1500 m (D), 2000 m (E) and 3000 m (F) in study area I. Filled and open circles denote forest patches with (*n* = 16) and without (*n* = 7) *M. rufocanus*, respectively. The *P*-values denote significance levels between groups as tested with Mann–Whitney *U*-test.

Focal forest patch size was significantly larger for patches with than without *M. rufocanus* in Västerbotten (*n* = 23; Fig. 5). Connectivity with surrounding forest patches was higher for focal patches with than without *M. rufocanus* for all the tested dispersal distances (250, 500, 1000, 2000 and 3000 m) and connectivity also increased with dispersal distance (Fig. 6A–F). The difference in connectivity between patches with and without *M. rufocanus* was clearer at larger distances and the variance in connectivity decreased with increased distance (Fig. 6A–F). Note that one outlier point has been removed from Fig. 6A–F.

In this study we have found that focal forest patch size, connectivity and occurrence of stone fields are positively related to vole occurrence. A discriminant analysis (Table 4) tested the relative importance of these variables to predict occurrence of *M. rufocanus* independently and in combination with forest patch size. Connectivity (250 m–3 km) was the best predictor variable for grey-sided vole presence and connectivity for 3 km dispersal distance also produced the best overall error rate of all tests (Table 4). Absence on the other hand was best predicted by a small area. Also, least-cost path distance (LCPdist) to stone fields was a better predictor for grey-sided vole presence than area alone. LCPdist combined with area and connectivity over 3 km showed the second lowest error value among all tested variable combinations and with better absence prediction than connectivity for 3 km dispersal distance alone (Table 4). Patch size and connectivity for different dispersal distances were also correlated to grey-sided vole *density* (*r* = 0.42–0.50; *P* < 0.05) but had lower *r*-values than the local structure variables large stone holes and pine forest (see above).

**Table 4:** Quadratic discriminant analysis of sampling plots with (*n* = 16) and without (*n* = 7) *M. rufocanus* in Västerbotten (see Fig. 2) for focal forest patch size (ha), connectivity (dIICflux/focal patch area) for distances of 250 m to 3 km and least-cost path distance to stone fields (LCPdist). Figures in brackets comes from the “leave-one-out” classification

Variable	Specificity (for MR = 0)	Sensitivity (for MR = 1)	Error rate
Area	1.0 (0.857)	0.625 (0.625)	0.261 (0.304)
250 m	0.571 (0.571)	1.0 (0.937)	0.130 (0.174)
500 m	0.714 (0.714)	1.0 (0.937)	0.087 (0.130)
1 km	0.714 (0.714)	0.937 (0.937)	0.130 (0.130)
1.5 km	0.571 (0.429)	1.0 (1.0)	0.130 (0.174)
2 km	0.714 (0.714)	0.937 (0.937)	0.130 (0.130)
3 km	0.857 (0.714)	1.0 (1.0)	0.043 (0.087)
Area + 250 m	1.0 (0.857)	0.687 (0.687)	0.217 (0.261)
Area + 500 m	0.857 (0.857)	0.687 (0.687)	0.261 (0.261)
Area + 1 km	0.857 (0.857)	0.687 (0.687)	0.261 (0.261)
Area + 1.5 km	1.0 (0.714)	0.687 (0.687)	0.217 (0.304
Area + 2 km	0.857 (0.714)	0.687 (0.687)	0.261 (0.304)
Area + 3 km	1.0 (0.714)	0.75 (0.75)	0.174 (0.261)
LCPdist	0.429 (0.429)	0.812 (0.812)	0.304 (0.304)
Area + LCPdist	1.0 (0.714)	0.75 (0.75)	0.174 (0.261)
Area + LCPdist + 3 km	1.0 (0.714)	0.812 (0.812)	0.130 (0.217)

## Discussion

### Dependence on habitat properties on microhabitat and local scale

The high frequency of occurrence of *M. rufocanus* in Västerbotten (I) compared to the almost complete absence in Norrbotten (II) demanded for the follow-up microhabitat inventory, which revealed that trap stations with *M. rufocanus* contained high numbers of large stone holes. This and the shorter distances to stone fields for sampling plots with *M. rufocanus* in Västerbotten compared to sampling plots in Norrbotten may partly explain the differences in *M. rufocanus* occurrence in the two study areas. In Västerbotten, high densities of grey-sided voles, directly linked to the number of large stone holes which provide shelter, were detected at several sampling plots (Fig. 4). This means that the species may be regarded as a forest- and stone specialist in lowland forests in Sweden, rather than a strict forest specialist as proposed by Kalela (1957). This is also in line with earlier findings for this species by Johannesen and Mauritzen (1999). Similarly, the snow vole (*Chionomys nivalis*) in the European Alps successfully uses holes in stone fields as shelter (see Yoccoz and Ims 1999).

### Dependence on habitat properties on the landscape scale

Christensen et al. (2008) and Ecke et al. (2010) found that occurrence of the grey-sided vole was dependent on large forest patch sizes. Here, we used patch size dependency in a landscape with high amount of old pine forest to successfully predict occurrence of grey-sided voles by trapping in a different study area (I) outside the long-term monitoring area in northern Sweden (Hörnfeldt 1994, 2004). This strengthens the focal forest patch hypothesis further. The number of replicates of large (>79 ha) lowland forest patches with grey-sided voles in the previous studies was low (*n* = 3 in both Christensen et al. 2008 and Ecke et al. 2010) while higher in area I in the current study (*n* = 16). In addition to patch size dependency, the discriminant analysis suggested that shorter distance to stone field components in the landscape positively influenced occurrence of *M. rufocanus* at the sampling plots (Table 4). To our knowledge, the positive effect of stone field components has not been quantified before.

Also, we have now for the first time shown that connectivity between forest patches also seems to be important for the grey-sided vole. Although our data set is too small to draw any firm conclusions on the relative importance of the tested predictor variables, the results suggest that connectivity might even be more important than area (Table 4). This strengthen the results by Christensen et al. (2008) which suggested that grey-sided voles are vulnerable to forest fragmentation due to clear-cutting. That study was largely based on populations that had shown a severe decline in numbers from the 1980s and onwards (see Hörnfeldt 2004). The surviving populations had probably retracted to some few source areas left in the landscape, that is, in contrast to the situation in our present study in area I. It is therefore reasonable to assume that this area is less fragmented than the one in Christensen et al. (2008).

Ecke et al. (2006) previously found that a higher proportion of clear-cuts in the surrounding matrix were negative for grey-sided vole persistence. The current results stress that the grey-sided vole is threatened by clear-cutting practices in boreal forest landscapes, both through the direct reduction in available forest habitat, but also through decreased connectivity between remnant forest patches. When the surrounding forest landscape (i.e., the matrix) is fragmented and contains large clear-cuts with grasses, the grass eating field vole *Microtus agrestis* (Siivonen 1968), may outcompete and suppress the grey-sided vole. *Microtus*-species are in most cases competitively superior to *Myodes* species, and in Kilpisjärvi, northern Finland, *M. rufocanus* retracted to nutrient poor areas in years when *M. agrestis* was abundant and occupied the mesic but not the nutrient poor sites (Viitala 1977). Also, clear-cuts pose a larger risk of predation by predators such as mustelids (Hansson 1994).

We propose two mechanisms explaining the positive effect of stone fields on grey-sided voles. Stone fields may (1) enhance matrix permeability for *M. rufocanus* by providing shelter from predators and (2) providing habitats both in forest patches and in the matrix. Based on our present data, we couldn't separate between these two separate positive effects of stone fields on grey-sided voles.

#### Forest management implications

It seems that it is of importance to the grey-sided vole, as to many other species, to concentrate forest protection efforts toward less fragmented parts of the landscape and to keep larger forest patches intact (Blake et al. 1984) and connected (Bergsten et al. 2013). According to Aune et al. (2006), this conservation practice should be adopted rather than saving scattered isolated forest islands in a landscape matrix dominated by clear-cuts. Old pine trees standing on coarse moraine soils are often left when harvesters clear-cut due to the difficulty of operating machines on stony ground. However, to our own experience, forest patches with intermediate content of stone fields are regularly clear-cut and followed by soil scarification before a new forest plantation is established. As discussed above, clear-cutting close to areas with stone fields may hamper grey-sided vole dispersal. Therefore, extra consideration in these situations may be required. An important future issue is to explore whether stone fields on clear-cuts can provide good quality habitats in themselves or merely provides shelter during dispersal between inhabited forest patches. For exploring this topic further, we will use time series on local vole dynamics and landscape changes to explore how connectivity between forest patches and stone fields has changed over time in northern Sweden and in relation to grey-sided vole occurrence and densities.
